# Making a Fire

**Published:** 1855-11

**Authors:** 


### MAKING A FIRE,

THESE cold November mornings, is a very necessary domestic item, and to do it certainly and quickly, will save more growls, and whines, and blessings "over the left," than the glibbest tongue could "*get over*" at a two-forty rate, in a year. Not only will it prove a saving of passion, but a saving of pence; for as it usually happens, the right way is the cheapest way in the end.

In the first place, if you are a bachelor or a maid, it is discreditable to you, if you do not kindle your own fires. What life it would infuse, how perfectly it would wake up a lazy sleeping child, if compelled to bounce out of bed at daylight of a winter's morning and light the anthracite! It sends the lazy sleeping blood to the remotest extremities, and quickens the whole body,—it vitalizes the man. *General Washington made it a practice to build his own fire at Mount Vernon;* and shame be on the young man or young woman, however rich the parents may be, who would feel it discreditable to kindle the fire of their own rooms.

### THE WAY TO DO IT.

Have your kindling wood cut not over five inches long, and split in pieces not larger than an inch square, but some of them should be mere splinters; take half a newspaper, and a quart or two of small coal or coke. These should be all placed near the grate over night; clean out the grate, at least the centre of it, crumple up the paper and lay it on the iron, set up the pieces of kindling in the shape of a tent or stack of arms, or an inverted funnel, the smaller splinters next the paper pressed closely against it, then lay the smaller pieces of coal, not much larger than the first joint of the thumb, close against the wood until the wood is hidden, then light a detached piece of paper with a match and place it under the grate, holding it close to the paper already there, let that paper fairly catch, put on the blower, and in about five minutes the coal will be ignited; then add one or two shovelsful more and replace the blower, and soon you will have a glowing fire without one failure in a whole winter; and it will not consume five minutes' time, after the grate is cleaned out.

But you must know the philosophy of all this, or you will not remember the details five minutes.

The wood must be small and in close proximity to the paper; for before anything burns, it must be saturated with caloric, it must get hot, and the smaller the piece of wood is, the sooner it will get hot, and the less heat, or caloric, will make it so; and as paper gives out but little heat, unless the wood is small, and close, it will be scattered, and thus fail to ignite. The same is particularly true of anthracite coal: it must be thoroughly heated before it takes fire, and it is easy to see, that it requires a less amount of caloric to heat a small piece of coal than a larger one, and less time, too;—thus it is, that the most effectual way of putting out a "poor" coal fire, is to fill up the grate with fresh coal; for there was enough caloric to have heated a few small pieces to the kindling point; but when distributed to a larger amount, none of it was raised to the degree requisite for ignition. *Therefore always put on a little coal at a time.*

In this way, as much wood four or five inches long, as may be grasped in one hand, is abundantly sufficient for kindling one fire promptly of anthracite coal, and certainly, thus we have kindled a fire two seasons with one load, that is, a third of a cord of pine wood. Families will economize by having the "lengths" theoretically four feet, practically, three and a half scant, cut six times; it gives more shillings to the sawyers, but fewer dollars to the wood-man. It will be of additional economy and interest to know, that in cleaning out the grate in the morning, you will have a good substitute for coke, if after separating the ashes, the pieces of partially burnt coal are thrown in a pail of water to be used next morning. They thus derive a new supply of oxygen from the water, and kindle easily with a bright flame. Whereas, if placed on the fire without having been soaked in water they moulder away, giving but little light or warmth. Only the black-looking pieces in the water are fit for burning again. If you do not have these, you must have coke, or use more wood.

